# Effectiveness of Prevention Interventions Using Social Marketing Methods on Behavioural Change in the General Population: A Systematic Review of the Literature

**DOI:** 10.3390/ijerph20054576

**Published:** 2023-03-04

**Authors:** Aude Roger, Mikael Dourgoudian, Virginie Mergey, David Laplanche, Fiona Ecarnot, Stéphane Sanchez

**Affiliations:** 1 Department of Performance and Public Health, Centre Hospitalier de Troyes, 10000 Troyes, France; 2 EA3920, University of Franche-Comté, 25000 Besançon, France; 3 Department of Cardiology, Besançon University Hospital, 25000 Besançon, France; 4 University Committee of Resources for Research in Health (CURRS), University of Reims Champagne-Ardenne, 51100 Reims, France

**Keywords:** social marketing, intervention, prevention, effectiveness, systematic review

## Abstract

In an effort to encourage people to adopt healthy behaviours, social marketing is increasingly used in disease prevention and health promotion. This systematic review aimed to evaluate the effect of prevention initiatives that use social marketing techniques on achieving behavioural change in the general population. We conducted a systematic review of PubMed, Embase, Science Direct, Cochrane, and Business Source Complete. Among 1189 articles identified across all databases, 10 studies met the inclusion criteria (six randomized controlled trials and four systematic reviews). The number of social marketing criteria used varies according to the studies. The results showed positive effects overall, albeit not always statistically significant. The quality of the studies was mixed: 3/4 of the systematic reviews did not meet the methodological criteria, and four out of six randomized trials had at least a high risk of bias. Social marketing is not fully exploited in prevention interventions. However, the greater the number of social marketing criteria used, the more positive the effects observed. Social marketing thus appears to be an interesting concept to bring about behavioural change, but it requires rigorous monitoring to ensure maximum effectiveness.

## 1. Introduction

Behavioural change and theories about individual behaviours have been widely employed in health promotion and in prevention campaigns [1,2,3]. In this context, social marketing has also been shown to be effective in enhancing the effectiveness of prevention or health promotion initiatives [1]. Indeed, according to the World Health Organization, behavioural risk factors are leading contributors to chronic diseases and their related morbidity and mortality [4]. Yet, a number of these risk factors are modifiable. Placing more emphasis on primary prevention could help to avert negative health outcomes and costly management, while at the same time improving the efficiency of healthcare pathways. In today’s context of scarce resources, it is vital to develop robust public health strategies based on programmes with proven efficacy. To this end, we perceived a need to evaluate the efficacy of interventions aimed at changing behaviours at population level.

Programmes designed to promote health and prevention are generally not designed to produce the marked effects in terms of disease outcomes in line with the methodological demands of evidence-based medicine. Health promotion or prevention campaigns that are deemed to have failed generally do so due to a misguided strategy, or because they did not reach a sufficient proportion of the target population [1]. Prevention campaigns are used to raise awareness among the general population about health issues, having the overarching aim of improving population health and wellbeing. However, the practical implementation of such programmes often faces methodological obstacles when trying to reconcile pragmatism with a high level of scientific evidence. Prevention programmes need to deliver an impactful message to encourage the target audience to adopt new behaviours, but at the same time, the evaluation of such programmes is not rigorously assessed using criteria established by evidence-based medicine [5]. 

Various tools can be used to promote the uptake of new health behaviours in the general population. Social marketing is one such tool that has been widely used to influence change in individual behaviours with methods that have been proven efficacious [6,7]. Since the emergence of the concept of social marketing, numerous definitions have been proposed. Initially defined as “the design, implementation, and monitoring of programs designed to influence the acceptability of social ideas” [8], the definition of social marketing has evolved over the years towards a more expansive one, in which marketing principles and techniques are applied to create, communicate, and deliver value in order to influence target audience behaviours that benefit society (public health, safety, the environment, and communities), as well as the target audience [6]. Summarizing the many available definitions, one might say that social marketing is the systematic application of techniques used for the benefit of individuals and society, rather than for purely commercial purposes [9,10,11]. Application of these marketing techniques in the domain of health requires criteria to judge their success. Several criteria have been defined and expanded over the years, and they include change in behaviour, as well as an understanding of the need, audience segmentation, exchange theory, marketing mix (price, produce, place, promotion), and competition. Other criteria have more recently been added [12,13,14]. The key principles of social marketing have been previously been described elsewhere [14]. 

In this context, prevention campaigns that apply social marketing techniques aim to modify attitudes, beliefs, and behaviours in terms of health, with a view of influencing individuals so that they voluntarily accept, reject, modify, or abandon certain behaviours [15,16]. A few papers have been published in the literature describing the evaluation of prevention programmes that applied social marketing in specific populations or diseases. However, no review to date has addressed the overall impact of social marketing techniques in healthcare prevention in the general population. Yet, it would be of major interest to understand how prevention initiatives that apply social marketing techniques bring about changes in behaviour in the population. Therefore, we aimed to perform a systematic review of the literature to evaluate the impact of prevention interventions that applied social marketing methods on achieving significant behavioural change in the general population of younger and older adults (14 to 65 years of age). 

## 2. Methods

We performed a systematic review of the literature in compliance with the PRISMA statement (Preferred Reporting Items for Systematic reviews and Meta-Analyses) [17] and the Cochrane Handbook for Systematic Reviews of Interventions [18]. The protocol for the review was registered with PROSPERO under the number CRD42022355147. 

### 2.1. Search Strategy

We searched the following databases: PubMed, Embase, Science Direct, Cochrane, and Business Source Complete. Using selected keywords and Boolean operators (AND, OR, NOT), to search the titles, abstracts and full text, we built a search strategy combining terms relating to social marketing, information, prevention, communication, health promotion, behaviour, and evaluation/assessment. The full details of the search strategy are provided in Supplementary Table S1. Two authors (AR, MD) designed and implemented the search strategy. 

### 2.2. Eligibility Criteria

We assessed all articles published in English since 2000 that resulted from the search (which started in April 2022), and included those that met the following eligibility criteria:

1. randomized, controlled trials, or systematic reviews;
2. use of social marketing as the predominant feature of the study; defined as “*a process that applies marketing principles and techniques to create, communicate and deliver value in order to infuence target audience behaviours that benefit society (e.g., public health, safety, the environment and communities) as well as the target audience*” [19];
3. with analysis of the efficacy of social marketing interventions (i.e., assessment of the effect of preventive interventions using social marketing techniques, on the willingness and/or ability to change potentially harmful and/or undesirable behaviours);
4. performed among the general population (aged 14–65 years).

### 2.3. Study Selection

After extraction of all search results, two authors (AR, MD) reviewed (blindly and independently) the titles and abstracts of all search results to assess them for eligibility for inclusion. The two lists were then compared and, in case of disagreement, a third author (SS) was consulted. 

The full text of all selected titles/abstracts were reviewed, and the reference lists were manually reviewed to ensure no relevant publications were overlooked. The final selection was made on the basis of the full text reading. 

### 2.4. Data Extraction

Data were extracted from the studies included and presented in the form of tables. In view of the different methodologies applied across studies, separate data extraction tables were created for the two main types of study (randomized studies and systematic reviews). For randomized trials, we extracted the title, authors, channels of communication used, objective(s) of the study, target(s), endpoint(s), measurement criteria, results, and bias. For systematic reviews, we extracted the title, authors, channels of communication used, objective(s) of the study, number of studies included, endpoint(s), measurement criteria, and results, and we added a column for the principles of social marketing that were used.

### 2.5. Assessment of Methodological Quality and Risk of Bias

Two authors assessed the methodological quality and the risk of bias for all studies included. For randomized studies, the quality was assessed using the updated RoB 2.0 tool from Cochrane [20], which evaluates potential for bias in five bias domains. In each domain, the answers to signalling questions help to reach an overall judgement about whether there is low risk of bias, some concerns or high risk of bias overall. If the study did not provide sufficient details to answer the signalling questions, we deemed there to be “some concerns” for that question. For systematic reviews, methodological quality was assessed using the AMSTAR 2 scale [21], which verifies whether each review meets 16 predefined quality criteria.

## 3. Results

**Figure 1:**
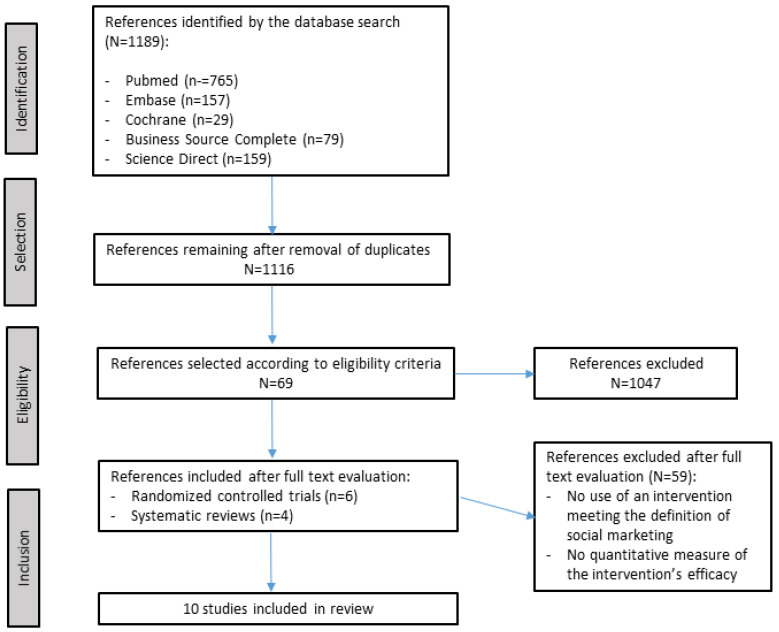
Flowchart of study selection.

The search yielded a total of 1189 articles. After removal of duplicates, we screened the titles and abstracts of 1116 articles, of which 69 were retained for full text reading. Cohen’s kappa coefficient for agreement between the two authors for inclusion of articles was 0.83. After full text reading, a final total of 10 studies [9,22,23,24,25,26,27,28,29,30] were included in the present review (six randomized trials and four systematic reviews). The flowchart of study selection is presented in Figure 1.

### 3.1. Results of Randomized Trials

A summary of the 6 randomized trials included [22,23,24,25,26,27] is presented in Table 1. 

Among the six trials included, various different methodologies were used, and two studies applied randomization in clusters [26,27].

Overall, four studies found a significant effect of the study intervention in terms of the desired behavioural change, while two did not. 

In a randomized trial, in which 30 worksite cafeterias were randomized to either the intervention, encouraging employees to choose healthier foods, or no intervention, Velema et al. reported that choice architecture (or “nudging”) had a significant positive effect for three of the seven food products studied, with a consistent increase in the purchase of the healthier options across the 12 weeks of the study intervention [22]. In a randomized trial among 468 competitive college athletes assigned to either a social marketing intervention about the possible consequences of reporting concussion or to one of two other control conditions, Warmath et al. reported that the intervention significantly increased positive beliefs, and it significantly decreased negative beliefs about the reporting of concussion [23].

DeJong et al. performed a randomized trial at 18 higher education institutions that were randomly assigned to the intervention or control groups [24]. The intervention consisted in social norms marketing campaigns aimed at changing perceptions of social drinking, with a view to ultimately reducing alcohol consumption. At the three-year post-intervention survey, the authors reported that the intervention campaign was significantly associated with lower perceptions of drinking levels and lower alcohol consumption. There was evidence to suggest a dose–response relationship between the intensity of the campaign and the magnitude of the reduction in drinking [24]. Finally, Stead et al. performed a randomized controlled trial among 53,367 participants, in which the intervention consisted in providing special offers on healthy foods, plus dietary advice and recipes among shoppers identified as making potentially “less healthy” food choices [25]. Among the 37,034 participants assigned to the intervention group, there was a significantly higher uptake of the promoted products during the month of the intervention, although the improvement was not sustained beyond the study period.

Two studies by Kamada et al. reported on the effects at three [26] and five years [27] of a community-wide intervention to promote physical activity. They randomly assigned 12 communities in Japan to either the intervention (aerobic activity promotion, flexibility and muscle-strengthening promotion, or promotion of a combination of both), or the control condition. The intervention was not found to result in a significant increase in the proportion of adults who reached recommended levels of aerobic, flexibility, and/or muscle-strengthening activities at three years [26]. At five years, adults achieving recommended levels of physical activities increased in the intervention communities, compared with the control [27]. Although the intervention was found to be effective in promoting all types of recommended physical activities, i.e., aerobic, flexibility and muscle-strengthening activities individually, a bundled approach, promoting all forms of activity simultaneously did not have a significant impact [27].

Regarding the methodological quality of the six randomized trials included in this review, four studies had a high risk of bias [22,23,24,25], and two studies had a low risk of bias [26,27] (Supplementary Table S2).

### 3.2. Results of Systematic Reviews

A summary of the four systematic reviews included [9,28,29,30] is presented in Table 2. 

Among the four reviews included, one investigated interventions promoting post-mortem organ donation, one examined the effect of social marketing in promoting healthy eating behaviours, and two focused on HIV. All four reviews reported positive effects, albeit not always statistically significant.

In a review of 32 articles describing 36 interventions in the USA, Canada, Italy, Colombia, Australia, and Sweden, Coz et al. reported that the interventions were generally very successful in achieving the defined goals, with a positive correlation observed between the number of social marketing benchmark criteria employed in the intervention, and the success rate of the interventions [28]. Indeed, interventions that used six or seven out of seven benchmark criteria reported success in all the intended goals, whereas interventions that employed fewer benchmark criteria had lower success rates.

In their review of social marketing to promote healthy eating behaviours, Carins et al. identified 18 studies that self-reported as social marketing studies, of which 16 presented at least five out of six criteria for social marketing studies [9]. The 16 studies that met the defining criteria were more successful in achieving behavioural change in terms of eating behaviours than studies that simply self-reported as social marketing studies, but without a clearly identifiable marketing orientation.

Two reviews focused on AIDS and HIV; one reviewed 19 studies investigating the effectiveness of mass media and communication interventions to increase HIV testing among men who have sex with men [29]. In this review, McDaid et al. found that six of the studies included reported increased HIV testing after the intervention. In the second review, Noar et al. conducted a 10-year systematic review of HIV/AIDS mass communication campaigns focused on sexual behaviour, HIV testing, or both [30]. From a total of 38 articles reporting 34 distinct campaigns in 23 countries, they additionally examined the 10 campaigns with the most methodologically rigorous design and found that eight of them demonstrated significant effects in changing behaviours (actual or intended).

### 3.3. Methodological Quality of Systematic Reviews

The methodological quality of the four systematic reviews is summarized in Supplementary Table S3.

## 4. Discussion

This review found that evidence that social marketing interventions may be successful in achieving changes in behaviour, although the magnitude of the effect was relatively small overall. Studies adhering to more criteria for social marketing achieved greater success. Despite methodological differences across the randomized trials included, significant effects were observed, with positive changes in behaviour. In one study [25], there was a significant increase in the proportion of customers purchasing healthier food products when these products were the focus of social marketing promotion. Another study used “nudging”, which is a choice architecture that attempts to alter behaviours without ruling out any choice options or changing financial incentives [22]. This technique, widely used in social marketing, helped to achieve a significant increase in the purchase of healthier food options in workplace canteens. Overall, the randomized studies included in this review did not all apply the full spectrum of social marketing criteria, which precludes any meaningful comparison of different social marketing approaches between studies.

The systematic reviews included in our review underline this point by showing that the studies applying the greatest number of social marketing criteria achieved the greatest success. Indeed, the studies in the reviews targeted change in a specific behaviour (e.g., HIV testing, choosing healthier foods, participate in organ donation), and most reported satisfactory rates of change in the desired behaviour. However, the number of social marketing criteria taken into consideration different across reviews, likely due to the progress in the definition of social marketing over time. The behavioural change criterion is included in almost all definitions, whereas the inclusion of theory is less common, and few studies integrated this criterion. The systematic reviews included here found that few interventions mobilized all the criteria for social marketing, with only one study out of 36 in the review of organ donation [28] and six out of 34 in the review of healthy eating behaviours [9]. Similarly, the communication methods used across all the interventions varied widely, although few, if any studies gave concrete details of the actual supports used to diffuse the messages. Consequently, it is unlikely that other teams could reproduce the interventions that were found to be effective in the absence of details about how exactly the intervention was implemented. 

### 4.1. Quality of the Studies

The quality of the studies included was judged to be low overall, with a high risk of bias. Therefore, the findings should be interpreted with caution. Among the systematic reviews, only one met all of the criteria in the AMSTAR II instrument for high quality. Among the randomized trials, there was potential for several forms of bias, including bias related to selection, attribution, and reporting. Selection bias can lead to a lack of representativeness, while attrition bias can affect the results due to patients being excluded or lost to follow-up. Bias in measurement and reporting can give a false or incomplete picture of the results. There are multiple reasons that could explain the presence of such potential for bias among the randomized trials. These include the randomization processes used, possible deviations from the planned interventions, missing data in the results, and judicious selection of the results to be presented. Among the six trials included here, four had a high risk of bias. The other two studies, reporting three- and five-year results from the same intervention, had a low risk of bias overall, but did not show any significant effect of the intervention. 

These findings highlight the challenges of applying the principles of evidence-based medicine to large-scale public health programmes and evaluation of such interventions. It is also notably challenging to measure changes in behaviour, which may be multifactorial in origin, and variable over time. Interventions aimed at changing behaviours need to be adapted to the specific context of the target population and the health determinants of that population. Health promotion requires involvement and communication that goes beyond the sole context of medical management. Change is a complex phenomenon, and the expected results depend on the interactions between a range of determinants, some of which may be beyond the control or reach of the intervention. Several mechanisms of action are possible to deal with this, but the application will only be optimal if the full spectrum of characteristics and needs of the population are taken into account, which is often unfeasible in practical terms. Finally, it is difficult to summarize behavioural change in a single criterion or metric, especially since the measure of change is itself subject to diverse definitions, notably as regards the timing and method of the measurement. Despite these potential drawbacks, this review shows that social marketing is a valuable tool for bringing about targeted behavioural change. Nevertheless, to exploit its full potential and yield maximum success, a guide to implementation is warranted to ensure systematic implementation based on validated criteria for success. Numerous theories have been proposed in recent decades regarding behavioural change and its measurement [31]. A meta-analysis of over 100 articles summarized data from the “fear appeal” literature and found that appealing to fear is an effective means to convey a persuasive message by wielding the spectre of a realistic threat to arouse fear [32]. The characteristics of the threat behind the message are at the heart of this model, whereby individuals who perceive the threat as improbable will ignore the message and will not be prompted to change behaviours. Conversely, if the target individuals perceive the threat to be credible and likely, their fear will prompt them to adopt the desired behaviour. When people feel that they have the wherewithal to act against the threat, then they will take appropriate action [32,33].

This study raises several perspectives. First, the use of social marketing techniques as a means to bring about behavioural change on various issues of social interest remains an emerging field, where there remains considerable scope for expansion. In this regard, the nascent nature of this field means that there is, as yet, a paucity of literature reporting quantitative evaluations on hard outcomes. Scientifically rigorous evaluation of the effects attributable to social marketing interventions are warranted to prove their efficacy, much in the same way as drugs and devices are submitted to extensive assessment before approval. To this end, the second perspective would be the development of a consensual definition of what constitutes a social marketing intervention in the field of health promotion and/or disease prevention, in order to harmonize studies and render comparison possible. The parallel use of quality indicators to verify the rigorous implementation of social marketing techniques is also warranted. This is line with the Plan-Do-Check-Act cycle recommended by the American Society for Quality (ASQ) for carrying out change. Indeed, in this approach, implementation of innovation is followed by monitoring and reporting to identify potential margin for improvement in a continuous quality improvement cycle. This approach could be implemented for health initiatives using social marketing techniques. Conversely, potential obstacles to the rigorous implementation include the diversity of definitions of “social marketing”, which hampers comparison across programmes; the heterogeneity in the health issues addressed as well as in the outcomes that may be assessed to quantify the improvements achieved. 

### 4.2. Study Limitations

This study has some limitations. Firstly, among 1187 articles identified, only 10 met our inclusion criteria. However, the use of social marketing techniques in the field of disease prevention and health promotion is an emerging practice, and as a result, there is a paucity of studies in the literature. Second, widely varying definitions of social marketing were used across all the studies, and the exact composition of the interventions varied, as did the level of detail of the descriptions of the interventions. This renders comparison between studies hazardous. Third, numerous other studies investigating the application of social marketing techniques are available, but they did not assess the efficacy of the interventions.

## 5. Conclusions

This study shows that few quantitative, randomized studies have investigated the efficacy of social marketing techniques in bringing about behavioural change in public health interventions. However, social marketing approaches are garnering increasing attention as a means to orient behaviours in the general population towards healthier options. Due to the complexity of implementing studies of this type, and the varying definitions employed, it is challenging to compare results between studies, and our review found a high risk of bias in many reports. Overall, the higher the number of quality criteria met by the social marketing intervention, the greater the success in terms of the expected behavioural change. Wider uptake of social marketing theory in the design of persuasive public health messages could help to achieve higher levels of desired behaviours. There is an unmet need for more training for public health and other healthcare professionals, as well as in the use and implementation of social marketing techniques in the field.

## Figures and Tables

**Table 1:** Summary of the randomized trials included in the review (N = 6).

Author, Country, Year [Ref]	Type of Communication	Target Population	Endpoint	Key Results
Velema, Netherlands, 2018 [22]	Nudging	An amount of 30 worksite cafeterias	Sales data for the targeted food products Compliance with the intervention protocol	Significant positive effect of the intervention on the volume of sales for three of the seven targeted product groups An amount of 77% of the eligible strategies were implemented correctly in the intervention cafeterias
Warmath, Greece, 2020 [23]	Three video interventions using three fact sheets (one for each of the three interventions)	An amount of 468 competitive athletes in a US university who engaged in 1 or 46 sports with concussion risk	Positive and negative beliefs regarding concussion reporting	Exposure to consequence-based social marketing significantly increased positive reporting beliefs and significantly decreased negative reporting beliefs as compared to the traditional or revised symptom-education conditions.
DeJong, United States of America, 2009 [24]	Social norms marketing campaigns about student drinking (norms and volume)	Cross-sectional survey in 2771 students at baseline and 2939 at three years in 18 higher education institutions	Perceptions of student drinking levels and level of alcohol consumption	Exposure to the SNM campaign was significantly associated with lower perceptions of student drinking levels and lower alcohol consumption. A moderate mediating effect of normative perceptions on student drinking was found. Suggestion of a dose-response relationship.
Stead, United Kingdom, 2017 [25]	Direct marketing price promotion combined with healthy eating advice and recipes for “less healthy” shoppers	An amount of 53,367 customers in a supermarket chain in he UK	Sales data from before, during and after the promotion	Increase in the proportion of customers buying promoted products in the intervention month for four out of five products. Significantly higher uptake in the promotion group in the intervention group compared to the expected average based on other months. Effects were not sustained beyond the intervention period.
Kamada, Japan, 2015 [26]	Community-wide intervention with cluster randomization using flyers, leaflets, posters, newsletters, banners and radio, plus outreach health education during community events, plus social support.	Community dwellers aged 49 to 70 years in 12 Japanese communities	Change in regular aerobic, flexibility, and/or muscle strengthening activities, evaluated at the individual level.	No significant effect of the intervention on the proportion of adults who reached recommended levels of aerobic, flexibility, or muscle-strengthening activities at three years compared to controls.
Kamada, Japan, 2018 [27]	Community-wide intervention with cluster randomization using flyers, leaflets, posters, newsletters, banners and radio, plus outreach health education during community events, plus social support.	Community dwellers aged 49 to 70 years in 12 Japanese communities	Change in regular aerobic, flexibility, and/or muscle strengthening activities, evaluated at the individual level.	At five years, compared to control communities, the proportion of adults achieving recommended levels of physical activity increased in intervention communities. The intervention was effective in promoting all three types of exercise individually, whereas a bundled approach promoting all three types simultaneously was not found to be effective. Pain intensity was found to decrease for the shoulder (in the intervention and control groups) and lower back (intervention group only).

**Table 2:** Summary of the randomized trials included in the review (N = 6).

Author [Ref]	Type of Communication	Target Population	Endpoint	Key Results
Coz, Slovenia, 2020 [28]	Mass media, multichannel approaches, direct (interpersonal face-to-face) communication	An amount of 32 articles describing 36 interventions promoting organ donation	Increasing donor registration rates was the most common primary behavioral objective of the interventions	There was a significant relationship between the use of social marketing benchmark criteria in the design of the intervention, and the success of the intervention. Interventions that employed six or seven (out of seven) criteria reported greater success in achieving intervention objectives.
McDaid, United Kingdom, 2019 [29]	Videos, television, radio, web-based advertisements, cinema and newspaper advertisements, posters, leaflets, etc.	An amount of 19 studies of interventions to increase HIV testing among men who have sex with men	Rate of HIV testing	Five cross-sectional and one randomized trial reported increased HIV testing after the intervention, providing evidence for the effectiveness of social marketing/mass media interventions to increase HIV testing. Risk of bias was high.
Carins, Australia, 2014 [9]	Social marketing processes incorporating individual and social activities	An amount of 34 empirical studies that self-reported as social marketing healthy eating interventions	Presence of social marketing benchmark criteria	Among sixteen studies with social marketing as a planned, consumer-oriented process, only six met all six benchmark criteria; the mean number of criteria was five. These 16 studies that met the definition of social marketing were more effective in achieving behavioural change than the 18 studies that self-reported as social marketing, but did not meet social marketing criteria.
Noar, United States of America, 2009 [30]	Mass communication campaigns focused on sexual behaviour, HIV testing, or both.	An amount of 38 HIV/AIDS campaign evaluation articles describing 34 distinct campaigns in 23 countries	Campaign design, and evaluation measures (change in behaviours or intentions)	There has been increasing use of the several strategies over time, including targeting defined audiences; designing campaigns around changes in behaviour (not knowledge); use of behavioural theories; high message exposure; stronger research design and measure of behavioural change (or intentions) as outcome assessments.

## Data Availability

Not applicable.

## Supplementary Materials

The following supporting information can be downloaded at: https://www.mdpi.com/article/10.3390/ijerph20054576/s1, Supplementary Table S1: full details of the search strategy; Supplementary Table S2: methodological quality of the 6 randomized trials included in this review; Supplementary Table S3: methodological quality of the 4 systematic reviews included in this review.
