# Supplementary material for: Effectiveness of Prevention Interventions Using Social Marketing Methods on Behavioural Change in the General Population: A Systematic Review of the Literature
Source: Int J Environ Res Public Health. 2023 Mar 4;20(5):4576. doi: 10.3390/ijerph20054576 (PMC10002445; doi:10.3390/ijerph20054576)
Supplement: Supplementary file 1 [file ijerph-20-04576-s001.zip › ijerph-2189306-supplementary.pdf]

# Supplementary Material

**Supplementary Table S1 : Search strings for each database**

| Database | Search string |
|---|---|
| **PubMed** | ((((("social marketing") OR (marketing of health services)) AND (((("consumer health information") OR (primary prevention)) OR (secondary prevention)) OR ("health promotion"))) AND (((health behavior) OR (health communication)) OR (persuasive communication))) AND (((program evaluation) OR (evaluation)) OR (effectiveness))) NOT (birth cohort OR child OR infant) ***(1)*** |
| **Embase** | Sources : Embase, MEDLINE, Preprints<br>Query : 'social marketing'/exp AND ('primary prevention'/exp OR 'consumer health information'/exp OR 'health promotion'/exp) AND ('health behavior'/exp OR 'medical information'/exp OR 'persuasive communication'/exp) AND ('program evaluation'/exp OR 'evaluation study'/exp OR effectiveness) AND 'adult'/exp NOT child |
| **Cochrane** | "marketing of health services" OR "social marketing" in All Text AND "Consumer health information" OR "Primary prevention" OR "Health promotion" in Title Abstract Keyword AND "Health behavior" OR "Health communication" OR "Persuasive communication" in Title Abstract Keyword AND "Program evaluation"OR "Evaluation" OR "Effectiveness" in Title Abstract Keyword AND "Adult" in Title Abstract Keyword - (Word variations have been searched) |
| **Business Source Complete** | ( SU "social marketing" AND ( health or healthcare or health care or medical ) AND ( consumer behavior or consumer attitudes ) ) NOT ( children or youth or child ) |
| **Science direct** | Find articles with these terms : "social marketing"<br>Title, abstract or author-specified keywords :<br>behavior AND (prevention OR health promotion OR campaign) AND (evaluation OR effectiveness OR efficiency) NOT (children OR child) |

***(1)****(("social marketing"[All Fields] OR ("marketing of health services"[MeSH Terms] OR ("marketing"[All Fields] AND "health"[All Fields] AND "services"[All Fields]) OR "marketing of health services"[All Fields])) AND ("consumer health information"[All Fields] OR ("primary prevention"[MeSH Terms] OR ("primary"[All Fields] AND "prevention"[All Fields]) OR "primary prevention"[All Fields]) OR ("secondary prevention"[MeSH Terms] OR ("secondary"[All Fields] AND "prevention"[All Fields]) OR "secondary prevention"[All*

*Fields]) OR "health promotion"[All Fields]) AND ("health behaviour"[All Fields] OR "health behavior"[MeSH Terms] OR ("health"[All Fields] AND "behavior"[All Fields]) OR "health behavior"[All Fields] OR ("health communication"[MeSH Terms] OR ("health"[All Fields] AND "communication"[All Fields]) OR "health communication"[All Fields]) OR ("persuasive communication"[MeSH Terms] OR ("persuasive"[All Fields] AND "communication"[All Fields]) OR "persuasive communication"[All Fields])) AND ("programme evaluation"[All Fields] OR "program evaluation"[MeSH Terms] OR ("program"[All Fields] AND "evaluation"[All Fields]) OR "program evaluation"[All Fields] OR ("evaluability"[All Fields] OR "evaluate"[All Fields] OR "evaluated"[All Fields] OR "evaluates"[All Fields] OR "evaluating"[All Fields] OR "evaluation"[All Fields] OR "evaluation s"[All Fields] OR "evaluations"[All Fields] OR "evaluative"[All Fields] OR "evaluatively"[All Fields] OR "evaluatives"[All Fields] OR "evaluator"[All Fields] OR "evaluator s"[All Fields] OR "evaluators"[All Fields]) OR ("effect"[All Fields] OR "effecting"[All Fields] OR "effective"[All Fields] OR "effectively"[All Fields] OR "effectiveness"[All Fields] OR "effectivenesses"[All Fields] OR "effectives"[All Fields] OR "effectivities"[All Fields] OR "effectivity"[All Fields] OR "effects"[All Fields]))) NOT ("birth cohort"[MeSH Terms] OR ("birth"[All Fields] AND "cohort"[All Fields]) OR "birth cohort"[All Fields] OR ("child"[MeSH Terms] OR "child"[All Fields] OR "children"[All Fields] OR "child s"[All Fields] OR "children s"[All Fields] OR "childrens"[All Fields] OR "childs"[All Fields]) OR ("infant"[MeSH Terms] OR "infant"[All Fields] OR "infants"[All Fields] OR "infant s"[All Fields])*

**Supplementary Table S2 : Assessment of the Risk of Bias in the Randomized Studies (N=6) using the updated Cochrane RoB tool**

| Domaines | Elaboration | Velema [20] | Warmath [21] | DeJong [22] | Stead [23] | Kamada [24,25] |
|---|---|---|---|---|---|---|
| **Bias arising from the randomization process** | Was the allocation sequence random? | Yes | No | Yes | PY/NI | Yes |
| | Was the allocation sequence concealed until participants were enrolled and assigned to interventions? | Yes | Yes | Yes | NI | Yes |
| | Did baseline differences between intervention groups suggest a problem with the randomization process? | No | PY | No | NI | PN |
| | **Risk of bias judgement** | Low | Some concerns | Low | Some concerns | Low |
| **Bias due to deviations from intended interventions** | Were participants aware of their assigned intervention during the trial? | Cafeterias yes, participants no | NI | Yes | No | No |
| | Were carers and people delivering the interventions aware of participants' assigned intervention during the trial? | Yes | No | Yes | No | PY |
| | *If Y/PY/NI to the two previous questions: Were there deviations from the intended intervention that arose because of the trial context?* | PN | PN | PN | NI | PN |
| | *If Y/PY/NI to previous question: Were these deviations likely to have affected the outcome?* | NI | NI | NI | NI | NI |
| | *If Y/PY to previous question: Were these deviations from intended intervention balanced between groups?* | NI | NI | NI | NI | NI |
| | Was an appropriate analysis used to estimate the effect of assignment to intervention? | Yes | Yes | Yes | No | Yes |
| | *If N/PN/NI to previous question: Was there potential for a substantial impact (on the result) of the failure to analyse participants in the group to which they were randomised?* | NI | NI | NI | No | Ni |
| | **Risk of bias judgement** | Low | Low | Low | Some concerns | Low |

| | | | | | | |
|---|---|---|---|---|---|---|
| **Bias due to missing outcome data** | Were data for this outcome available for all, or nearly all, participants randomised? | Nearly all | NI | Ni | NI | Yes |
| | *If N/PN/NI to previous question: Is there evidence that the result was not biased by missing outcome data?* | NI | No | No | No | NI |
| | *If N/PN to previous question: Could missingness in the outcome depend on its true value?* | NI | NI | NI | NI | NI |
| | *If Y/PY/NI to previous question: Is it likely that missingness in the outcome depended on its true value?* | NI | NI | NI | NI | NI |
| | **Risk of bias judgement** | Low | High | High | High | Low |
| **Bias in measurement of the outcome** | Was the method of measuring the outcome inappropriate? | PY | PN | PN | PN | NI |
| | Could measurement or ascertainment of the outcome have differed between intervention groups? | No | No | PY | PN | PN |
| | *If N/PN/NI to two previous questions: Were outcome assessors aware of the intervention received by study participants?* | Yes | No | Yes | Yes | No |
| | *If Y/PY/NI to previous question: Could assessment of the outcome have been influenced by knowledge of intervention received?* | No | NI | NI | PN | PN |
| | *If Y/PY/NI to previous question: Is it likely that assessment of the outcome was influenced by knowledge of intervention received?* | NI | NI | PY | NI | NI |
| | **Risk of bias judgement** | High | Low | High | Low | Low |
| **Bias in the selection of reported results** | Were the data that produced this result analysed in accordance with a prespecified analysis plan that was finalised before unblinded outcome data were available for analysis? | Yes | Yes | Yes | NI | Yes |
| | Is the numerical result being assessed likely to have been selected, on the basis of the results, from multiple eligible outcome measurements (eg, scales, definitions, time points) within the outcome domain? | PY | PN | PN | NI | PN |
| | Is the numerical result being assessed likely to have been selected, on the basis of the results, from multiple eligible analyses of the data? | NI | NI | NI | NI | PN |
| | **Risk of bias judgement** | Some concerns | Some concerns | Some concerns | Some concerns | Low |

| | | | | | | |
|---|---|---|---|---|---|---|
| **Overall result** | Low: low risk of bias for all domains. Some concerns: some concerns in several domains that considerable reduce confidence in the results. High: high risk in at least one domain. | High | High | High | High | Low |

PY, probably yes ; PN, probably no; NI, no information.

**Supplementary Table S3 : Methodological quality of the systematic reviews, as assessed by the AMSTAR-II tool**

| Items | Cotation | Coz [26] | McDaid [27] | Carins [9] | Noar [28] |
|---|---|---|---|---|---|
| **1** | Did the research questions and inclusion criteria for the review include the components of PICO? | No | Yes | No | Yes |
| **2** | Did the report of the review contain an explicit statement that the review methods were established prior to the conduct of the review and did the report justify any significant deviations from the protocol? | NI | Yes | NI | NI |
| **3** | Did the review authors explain their selection of the study designs for inclusion in the review? | Yes | Yes | Yes | NA |
| **4** | Did the review authors use a comprehensive literature search strategy? | Yes | Yes | Yes | No |
| **5** | Did the review authors perform study selection in duplicate? | NI | Yes | NI | Yes |
| **6** | Did the review authors perform data extraction in duplicate? | NI | Yes | NI | Yes |
| **7** | Did the review authors provide a list of excluded studies and justify the exclusions? | Yes | Yes | Yes | No |
| **8** | Did the review authors describe the included studies in adequate detail? | No | Yes | No | Yes |
| **9** | Did the review authors use a satisfactory technique for assessing the risk of bias (RoB) in individual studies that were included in the review? | No | Yes | No | No |
| **10** | Did the review authors report on the sources of funding for the studies included in the review? | No | Yes | No | No |

| | | | | | |
|---|---|---|---|---|---|
| **11** | If meta-analysis was performed did the review authors use appropriate methods for statistical combination of results? | NA | NA | NA | NA |
| **12** | If meta-analysis was performed, did the review authors assess the potential impact of RoB in individual studies on the results of the meta-analysis or other evidence synthesis? | NA | NA | NA | NA |
| **13** | Did the review authors account for RoB in individual studies when interpreting/ discussing the results of the review? | No | Yes | No | No |
| **14** | Did the review authors provide a satisfactory explanation for, and discussion of, any heterogeneity observed in the results of the review? | No | Yes | Yes | Yes |
| **15** | If they performed quantitative synthesis did the review authors carry out an adequate investigation of publication bias (small study bias) and discuss its likely impact on the results of the review? | NI | NI | NI | NI |
| **16** | Did the review authors report any potential sources of conflict of interest, including any funding they received for conducting the review? | Yes | Yes | Yes | No |

NA, Not applicable; NI, no information.
